# EIF2S3Y suppresses the pluripotency state and promotes the proliferation of mouse embryonic stem cells

**DOI:** 10.18632/oncotarget.7187

**Published:** 2016-02-04

**Authors:** Na Li, Hailong Mu, Liming Zheng, Bo Li, Chongyang Wu, Bowen Niu, Qiaoyan Shen, Xin He, Jinlian Hua

**Affiliations:** ^1^ College of Veterinary Medicine, Shaanxi Center of Stem Cells Engineering and Technology, Northwest A & F University, Yangling, Shaanxi, China

**Keywords:** EIF2S3Y, embryonic stem cells, pluripotency, proliferation, mouse

## Abstract

Eukaryotic translation initiation factor 2, subunit 3, and structural gene Y-linked (EIF2S3Y) is essential for spermatogenesis in mouse models. However, its effect on embryonic stem (ES) cells remains unknown. In our observation, differentiated ES cells showed higher levels of EIF2S3Y. To further elucidate its role in ES cells, we utilized ES-derived EIF2S3Y-overexpressing cells and found that EIF2S3Y down-regulated the pluripotency state of ES cells, which might be explained by decreased histone methylation levels because of reduced levels of ten-eleven translocation 1 (TET1). Moreover, EIF2S3Y-overexpressing cells showed an enhanced proliferation rate, which might be due to increased Cyclin A and Cyclin E levels. This study highlighted novel roles of EIF2S3Y in the pluripotency maintenance and proliferation control of ES cells, which would provide an efficient model to study germ cell generation as well as cancer development using ES cells, thus providing valuable target for clinical applications of ES cells.

## INTRODUCTION

Embryonic stem (ES) cells are derived from the inner cell mass of blastocyst-stage embryos; they can form any fully differentiated cells of the body because of their pluripotent nature [1]. It has been widely reported that ES cells can differentiate into ectodermal [2], mesodermal [3], and endodermal cells [4, 5]. To maintain the pluripotent state, core transcription factors, such as octamer-binding transcription factor (OCT4), sex-determining region Y-box 2 (SOX2) and nanog homeobox (NANOG), are required [6–8]. A delicate regulation of the crucial genes and corresponding transcriptional factors dictates the pluripotent state of ES cells [7–9]. Recently, it has also been proved that histone modification patterns are involved in these states [9, 10]. Previous studies have shown that TET1 is highly expressed in ES cells, and its expression level varies considerably between pluripotent and differentiated states [10]. Moreover, TET1 is also important in activating DNA demethylation [9, 11]. Another crucial factor, eIF-2, functions in the early steps of protein synthesis by forming a ternary complex with GTP and initiator tRNA [12]. It is widely known that eIF- 2 is composed of three subunits, α, β and γ. EIF2S3Y, the gene encoding eIF-2γ, was identified on the mouse Y chromosome [13]. Later, its essential role in mouse spermatogenesis was discovered [14–17]. Upon the transgenic addition of sex-determining region Y (SRY) and EIF2S3Y to male mice with an X chromosome but without a Y chromosome, a substantial number of spermatocytes complete the first meiotic division [18], with the occasional production of spermatid-like cells [14, 18]. Yamauchi et al. demonstrated that these spermatid-like cells were functional in assisted reproduction and that SRY could increase the development of functional gametes [15]. Thus, EIF2S3Y plays an important role in male reproduction.

Though EIF2S3Y is essential for the spermatogenesis in mouse models [14], its effect on ES cell has been rarely reported. In this study, we found that ES-derived cell lines overexpressing EIF2S3Y showed reduced pluripotency and a faster proliferation rate than ES cells. Our results indicated that altered TET1 and 5hmC levels and histone methylation patterns might account for the pluripotency difference. Moreover, increased Cyclin A and Cyclin E levels might explain why EIF23SY promoted proliferation. Our results for the first time demonstrated the role of EIF2S3Y in ES cell pluripotency maintenance and proliferation control, which may provide help for clinical applications of ES cells.

## RESULTS

### Generation of the ES-derived cell lines

**Figure 1:**
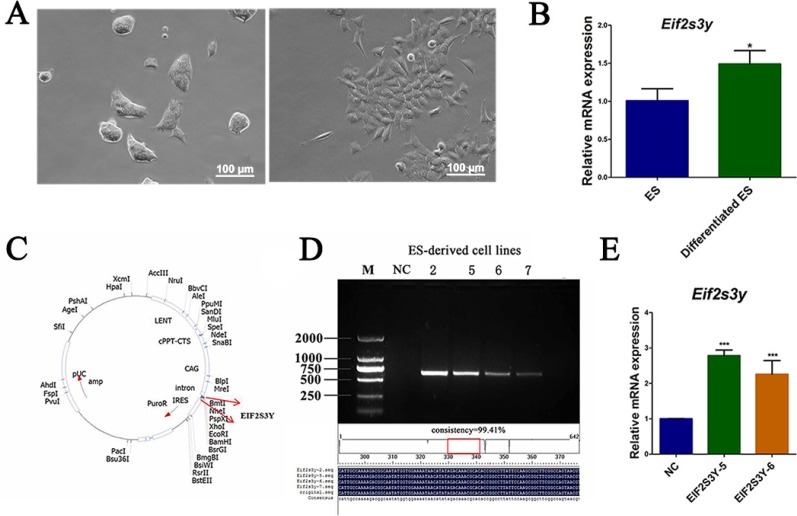
The ES-derived cell lines were established (**A**) ES cells were cultured in ES culture medium supplemented with (left) or without (right) RA for 48 h, the morphology was illustrated. (**B**). *Eif2s3y* mRNA level of untreated or RA treated ES cells. (**C**) Construction of lentivirus expression vector containing EIF2S3Y gene. (**D**) Generation of the cell lines EIF2S3Y-2, 5, 6, and 7. (**E**) Relative *Eif2s3y* mRNA expression analyzed by qRT-PCR (*n* = 3 for each group, *P < 0.05 vs. ES, ***P < 0.001 vs. NC).

To elucidate the role of EIF2S3Y in ES cells, we analyzed its expression levels in untreated ES cells and ES cells treated by retinoic acid (RA) for induced differentiation. After differentiation by RA for 48 h (Figure 1A), we found that these differentiated ES cells showed a significantly higher level of *Eifs3y* compared with untreated ES cells (Figure 1B). Considering this, and to better explore the function of EIF2S3Y, we cloned *Eif2s3y* by PCR from adult mouse testes, and then constructed the recombination plasmid *pTRIP-CAGG*-*Eif2s3y* (Figure 1C). ES cells were transduced with the lentivirus pTRIP-CAGG-*Puro* and pTRIP-CAGG-*Eif2s3y*, and then cultured with the ESGRO Complete PLUS Clonal Grade Medium supplemented with puromycin. Approximately 2 weeks later, we plated the cells into 96-well plates (one cell per well) using the limiting dilution method and finally obtained 7 ES-derived cell lines, named EIF2S3Y-1 to -7, respectively. ES cell line infected with only pTRIP-CAGG-*Puro* was used as a negative control (NC). To evaluate whether the cells were successfully transduced by EIF2S3Y, we examined the genomic integration of the exogenous genes and detected 4 positive cell lines: EIF2S3Y-2, EIF2S3Y-5, EIF2S3Y-6 and EIF2S3Y-7 (Figure 1D). EIF2S3Y-5 and EIF2S3Y-6 were used in this study because they exhibited the highest EIF2S3Y mRNA levels (Supplementary Figure 1 and Figure 1E).

### Reduced pluripotency of EIF2S3Y-5 and EIF2S3Y-6 cell lines

**Figure 2:**
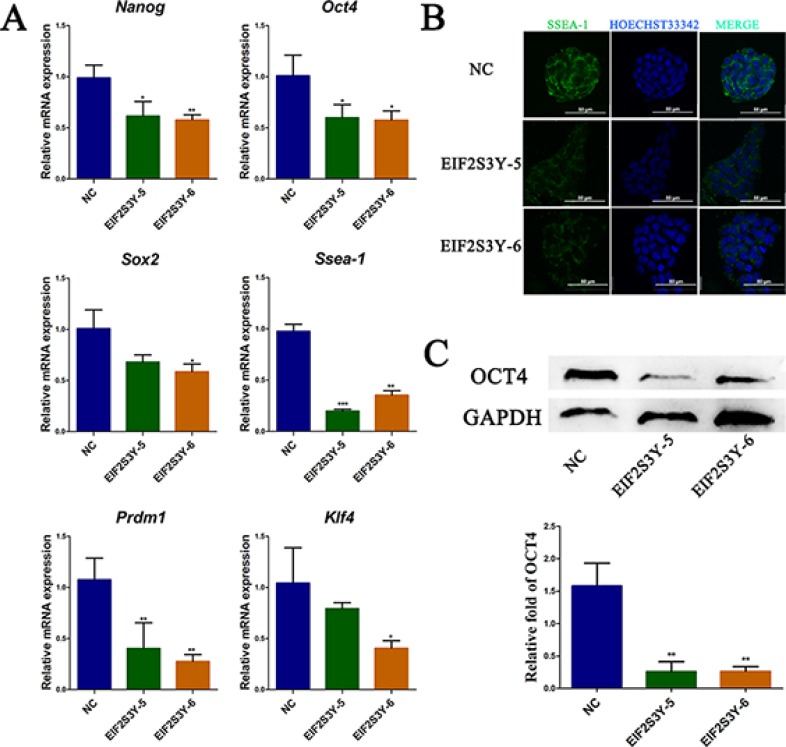
Pluripotency analysis of ES-derived cell lines (**A**) Expression of pluripotency associated genes *Nanog, Oct*4, *Sox*2, *Ssea*-1, *Prdm*1, and *Klf*4, measured by qRT-PCR. (**B**) Immunofluorescence staining of SSEA-1 (green), nucleic acids were stained with Hoechst33342 (blue). Scale bar = 50 μm. (**C**) Western blot and related densitometric analysis of OCT4 in NC, EIF2S3Y-5, and EIF2S3Y-6 cell lines (*n* = 3 for each group, *P < 0.05, **P < 0.01 vs. NC).

It is widely known that several transcription factors and specific markers, including SSEA-1, OCT4, SOX2 and NANOG, are required for maintaining ES cell pluripotency [7, 19]. Interestingly, we observed a significant decrease in SSEA-1 mRNA and protein levels in EIF2S3Y-5 and EIF2S3Y-6 cell lines compared with NC (Figure 2A, 2B). Meanwhile, the core transcription factors including *Nanog*, *Oct4*, and *Sox2*, which governed the pluripotent state [7], were down-regulated in EIF2S3Y-5 and EIF2S3Y-6 cell lines (Figure 2A, 2C). Moreover, we also found decreased levels of *PR domain zinc finger protein 1 (PRDM1)* and *kruppel-like factor 4 (KLF4)*, as expected (Figure 2A). Furthermore, decreased levels of OCT4 and SOX2 were detected by immunofluorescence staining in EIF2S3Y-5 and EIF2S3Y-6 cells (Supplementary Figure 2A–2B).

### Reduced differentiation ability of EIF2S3Y-5 and EIF2S3Y-6 cell lines

**Figure 3:**
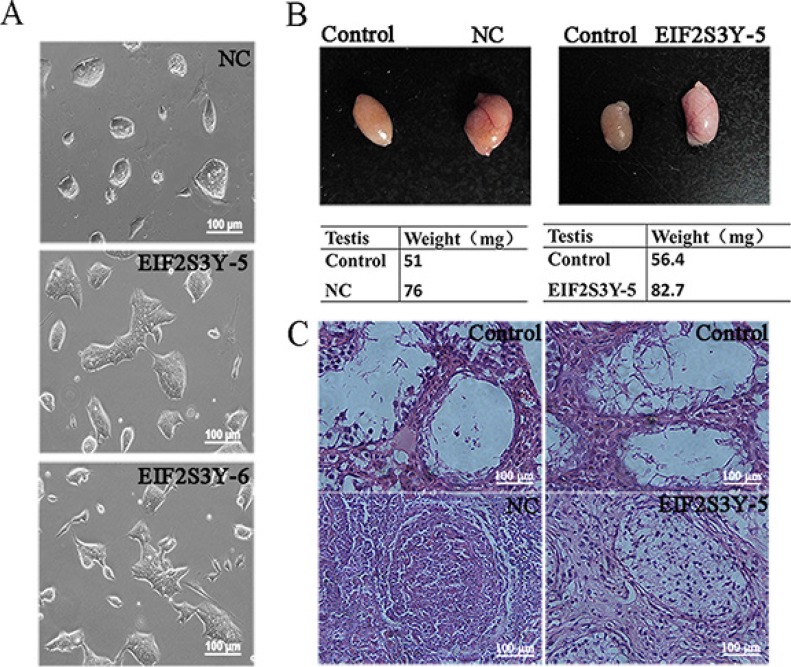
Differentiation ability analysis of ES-derived cell lines (**A**) Morphology of NC, EIF2S3Y-5, and EIF2S3Y-6 cell lines. (**B**) Morphology and weight of the testes transplanted (right) and not transplanted (left) with NC or EIF2S3Y-5 cell lines. (**C**) H & E staining of the testes transplanted (down) or not transplanted (up) with NC or EIF2S3Y-5 cell line. Scale bar = 100 μm (*n* = 2 for each group).

We compared the morphology of EIF2S3Y-5 and EIF2S3Y-6 cell lines with that of ES cells, and found that while ES cell clones showed a compact, round-shaped morphology, the EIF2S3Y-overexpressing cell clones became relatively loose and elongated (Figure 3A), indicating that these EIF2S3Y-overexpressing cells were differentiated to some extent, further proved our previous observations that these cells had reduced pluripotency. To further confirm this, we transplanted ES and EIF2S3Y-5 cells into the seminiferous tubules of germ cell-deficient infertile mice, considering that EIF2S3Y is indispensable for spermatogenesis [16]. Our results showed that there was an increase in both the volume and weight in NC- and EIF2S3Y-5- transplanted testis (Figure 3B). However, hematoxylin and eosin staining showed that while the testis transplanted with ES-NC cells formed typical teratomas, the testis transplanted with EIF2S3Y-5 generated only germ cell-like cells in seminiferous tubules (Figure 3C, and Supplementary Figure 3). Hence, we suggested that the EIF2S3Y-overexpressing cells had reduced differentiation ability compared with ES cells.

### Increased TET1 and decreased histone methylation levels in EIF2S3Y-5 and EIF2S3Y-6 cell lines

**Figure 4:**
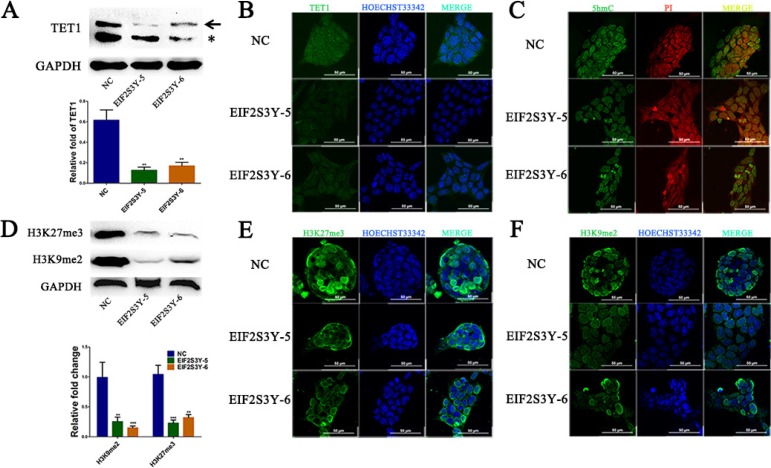
TET1 expression levels and histone methylation patterns in ES-derived cell lines (**A**) Western blot analysis of TET1 in NC, EIF2S3Y-5, and EIF2S3Y-6 cell lines. The arrow indicates the anticipated band of TET1, and the asterisk indicates a non-specific band. (**B**) Immunofluorescence staining of TET1 in NC, EIF2S3Y-5 and EIF2S3Y-6 cell lines. Scale bar = 50 μm. (**C**) Immunofluorescence staining of 5hmC in NC, EIF2S3Y-5 and EIF2S3Y-6 cell lines. Scale bar = 50 μm. (**D**) Western blot and related densitometric analysis of H3K27me3 and H3K9me2 in NC, EIF2S3Y-5 and EIF2S3Y-6 cell lines. (**E, F**) Immunofluorescence staining of H3K27me3 and H3K9me2 in NC, EIF2S3Y-5 and EIF2S3Y-6 cell lines. Scale bar = 50 μm (*n* = 3 for each group, **P < 0.01, ***P < 0.001 vs. NC).

TET1 has an important role in the self-renewal and maintenance of ES cells, especially in the 5mC to 5hmC conversion [20]. In fact, various analyses showed that ES cells had high TET1 levels (Figure 4A, 4B), which was in agreement with a previous report [11]. In contrast, significantly weaker TET1 signals were detected in EIF2S3Y-5 and EIF2S3Y-6 cell lines, and TET1 was localized mostly in the nuclei (Figure 4A, 4B). It has been reported that TET1 and other TET family members are required for 5hmC generation in ES cells under physiological conditions [21]. Therefore, we hypothesized that the down-regulation of TET1 might result in a parallel decrease in 5hmC levels. As expected, a higher expression level of 5hmC was detected in NC compared with relatively lower levels in EIF2S3Y-5 and EIF2S3Y-6 cells (Figure 4C). Moreover, it was reported that decreased TET1 could resulted in a change in histone methylation pattern, which also affected the pluripotent state of ES cells [22–24]. Among these epigenetic modifications, H3K9me2 and H3K27me3 are two crucial factors for the repression of ES cell differentiation [25–27]. We next performed western blot assays and immunofluorescence staining with antibodies specific to H3K27me3 and H3K9me2. As expected, we found their levels were both decreased in EIF2S3Y-overexpressing cells (Figure 4D–4F). H3K27me3 was distributed uniformly throughout the nuclei in undifferentiated ES cells [28], whereas it exhibited a specific peri-nuclear distribution pattern in EIF2S3Y-5 and EIF2S3Y-6 cells (Figure 4E). In contrast, H3K9me2 was localized throughout the nuclei in all 3 groups of cells (Figure 4F). These results indicated that EIF2S3Y down-regulated the level of TET1, which further decreased the level of 5hmC and histone epigenetic modifications, thus reducing the pluripotent state of ES cells.

### Increased cell proliferation rate in EIF2S3Y-5 and EIF2S3Y-6 cell lines

**Figure 5:**
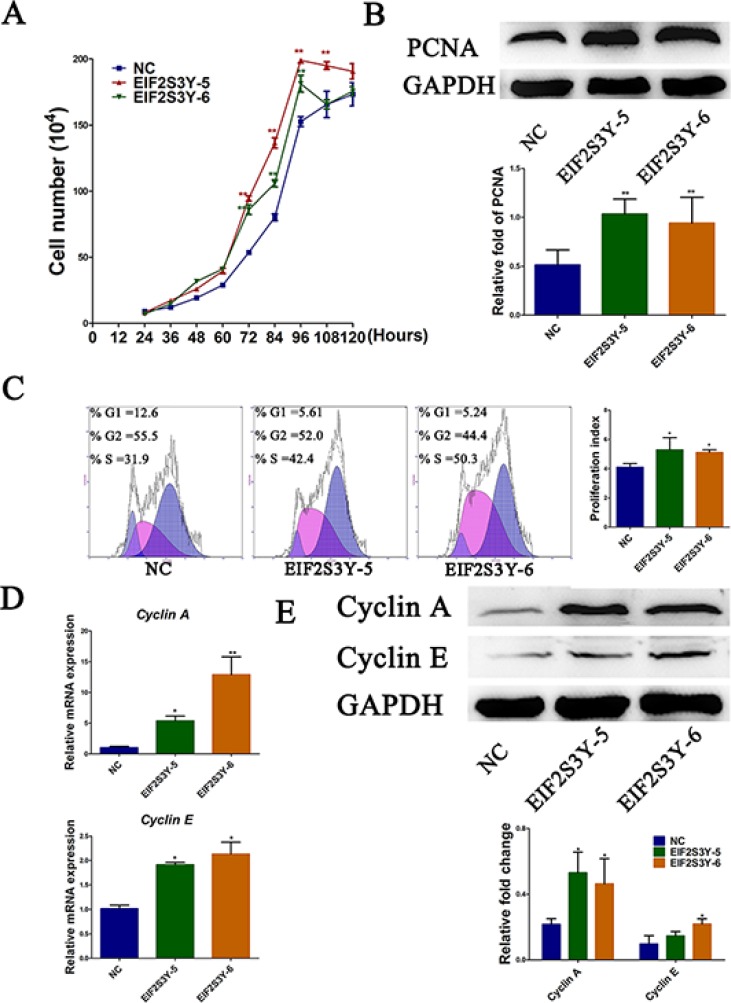
Proliferation profile of ES-derived cell lines (**A**) Growth curve of NC, EIF2S3Y-5 and EIF2S3Y-6 cell lines. (**B**) Western blots and related densitometric analysis of PCNA in NC, EIF2S3Y-5, and EIF2S3Y-6 cell lines. (**C**) Cell cycle analysis of NC, EIF2S3Y-5, and EIF2S3Y-6 cell lines by flow cytometry and related proliferation index. (**D**) Relative expression of Cyclin A and Cyclin E by qRT-PCR in NC, EIF2S3Y-5 and EIF2S3Y-6 cell lines. (**E**) Western blot and related densitometric analysis of Cyclin A and Cyclin E in NC, EIF2S3Y-5 and EIF2S3Y-6 cell lines (*n* ≥ 3 for each group, *P < 0.05 vs. NC).

Proliferation ability is crucial for the self-renewal of ES cells, and the self-renewal profile of ES cells is of great importance in regenerative medicine nowadays. Thus, we next analyzed the role of EIF2S3Y in ES cell proliferation. In fact, we noticed the EIF2S3Y-overexpressing cell lines proliferated more vigorously during cell culture. To quantify this observation, various methods, including cell number counting, PCNA immunoblotting, BrdU immunostaining, and flow cytometry, were used, and all these results confirmed our observation that EIF2S3Y-5 and EIF2S3Y-6 cells had higher proliferation rates (Figure 5A–5C, Supplementary Figure 4A–4C). It is widely reported that Cyclin A and Cyclin E are both crucial for cell proliferation [29–32]. Therefore, we next asked whether this increased proliferation profile was due to increased levels of Cyclin A and Cyclin E. In fact, EIF2S3Y overexpression resulted in significantly higher levels of both Cyclin A and Cyclin E (Figure 5D, 5E). In aggregate, our results demonstrated a previously unknown role for EIF2S3Y in promoting the proliferation of ES cells.

## DISCUSSION

The EIF2S3Y gene, which is Y-linked and associated with spermatogenesis, is conserved in the *Rhesus monkey*, *cow*, *M.oryzae*, *N.crassa*, and *A.thaliana*. Previous studies have demonstrated that live mouse progeny can also be generated using germ cells from males with the Y chromosome contribution limited to only two genes, *Sry* and the spermatogonial proliferation factor *Eif2s3y* [15]. It was later suggested that spermiogenesis could be initiated regardless of whether a second meiotic division has occured or not [16], and even when the only Y genes present are EIF2S3Y and SRY [15]. Recently, studies of Yamauchi group showed that only three Y chromosome genes, *Sry*, *Eif2s3y* and *Zfy2*, constituted the minimum Y chromosome complement compatible with successful intracytoplasmic sperm injection in the mouse [33].

TET1 is highly expressed in ES cells and iPS cells and plays an important role in activating DNA demethylation [9, 11]. The state of stem cells is regulated by TET1 and histone methylation patterns [10, 11]; especially, TET1 has an important role in ES cell self-renewal and maintenance [20]. TET1 has dual functions in the transcriptional regulation of mouse ES cells, activating transcription of pluripotency factors and participating in repression of polycomb-repressed genes [12]. Its function in ES cells is mediated through maintaining NANOG expression to some extent [20], and ES cell differentiation is accompanied by a decrease of TET1 level [34]. Moreover, epigenetic modification is also of great importance for ES cell pluripotency maintenance. It has been reported that H3K27me3 levels are significantly decreased in neural precursor cells compared with ES cells [23, 35]. Our study showed that EIF2S3Y down-regulated TET1 expression, which decreased the histone methylation patterns and reduced the pluripotency of ES cells as a result. Therefore, we suggest that TET1 might be the key factor controlled by EIF2S3Y to affect the pluripotency of ES cells.

Generally speaking, proliferation ability is closely associated with the differentiation potentiality of ES cells. However, it is unsurprising that reduced pluripotency and increased proliferation rate appear at the same time. In a study of periodontal ligament stem cells, it was reported that periodontitis could promote the proliferation and suppress the differentiation potential of periodontal ligament stem cells [36]. Which highlighted that increased proliferation and decreased differentiation ability could co-exist. Besides, it has already been reported that some factors could have opposing roles in terms of pluripotency maintenance and proliferation ability, such as MEK/ERK signaling pathway [37–44]. Moreover, KLF4 could be a tumor suppressor protein limiting cell growth [45, 46]. Accordingly, the mechanism of our study might involve, but not limited to, the signaling of MEK/ERK and KLF4. In fact, we detected reduced level of KLF4 after EIF2S3Y overexpression (Figure 2A). Even though we need more evidences to support this hypothesis, we believe a likely mechanism may exist, and we will continue to explain this conclusion more deeply next.

Considering clinical applications, pluripotency and self-renewal of ES cells have made them very promising regarding their potential use in regenerative medicine. However, these unique features of ES cells could also make them dangerous. That is because tumors, including teratomas, or more malignant, teratocarcinomas, would be genereated when applied *in vivo* [47–50]. In fact, tumorigenesis is the most challenging problem that hinders ES cells' clinical application.

The origins and fates of cancer are still a fascinating black box. The cells of the germ line, primitive germ cells (i.e. primordial germ cells) arise in the wall of the yolk sac, which are highly motile and could migrate to the gonadal primordium [51]. This process of germ cell colonization of the gonad in many ways resembles the progression of cancer cells from primary tumor to metastasis [52]. Moreover, during spermatogenesis, germ cells exhibit characteristics similar to cancer cells [52].

In our *in vivo* study, when transplanted into the seminiferous tubules of germ cell-deficient infertile mice, EIF2S3Y-overexpressing ES cells did not generate teratomas as normal ES cells did, but rather some of them differentiated into VASA positive germ cell-like cells (Supplementary Figure 3). So, our results turned out to be that even though EIF2S3Y-overexpression decreased pluripotency of ES cells to some extent, these cells still differentiated into germ cell-like cells in the seminiferous tubules. Most importantly, no teratomas would be generated during this process, which would make it safer if they were used to cure diseases such as infertility. Moreover, during cell number amplification *in vitro* if they were to be used clinically, these EIF2S3Y-overexpressing ES cells would proliferate more vigorously than normal ES cells. Thus, our work could provide potentially valuable target for the clinical application of ES cells.

**Figure 6:**
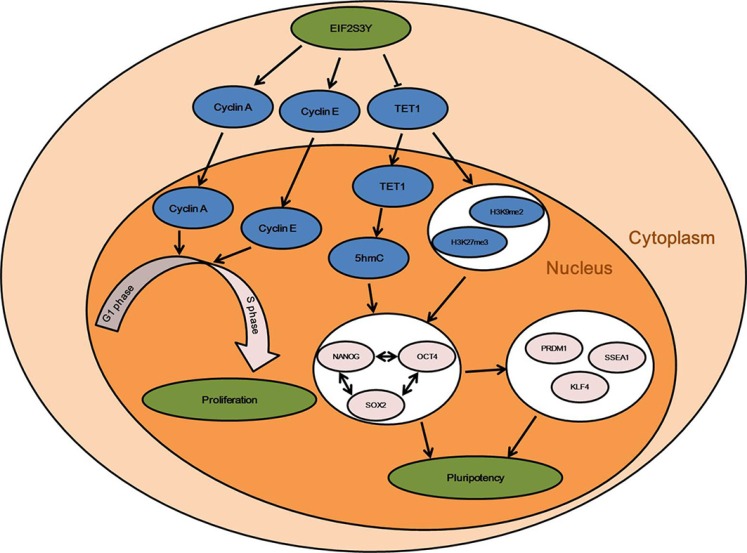
Effects of EIF2S3Y on pluripotency maintenance and proliferation rate of mouse ES cells EIF2S3Y in ES cells inhibited the expression of TET1, which governs the pluripotent state of ES cells by maintaining key factors such as NANOG, OCT4, SOX2, PRDM1, SSEA1, and KLF4, through 5hmC conversion and histone epigenetic modifications. Moreover, EIF2S3Y can also stimulate the proliferation profile of ES cells by helping the translation of Cyclin A and Cyclin E. Thus, EIF2S3Y expression in ES cells influences both their pluripotency state and proliferation profile of ES cells.

Collectively, we found EIF2S3Y-overexpression downregulated Tet1 level in mouse ES cells. Consequently, decreased Tet1 level resulted in the reduced pluripotency of these ES cells. This is underpinned by down-regulated Nanog, Oct4, Sox2, Prdm1, Ssea1, and Klf4 levels in the EIF2S3Y-overexpressing ES cells, which could all be regulated by Tet1. Moreover, epigenetic modifications, which affected ES cell pluripotency, and which could also be affected by Tet1, changed after EIF2S3Y-overexpression. Besides, EIF2S3Y-overexpression could also stimulate the proliferation of ES cells, partly through up-regulating Cyclin A and Cyclin E (Figure 6). Our study for the first time highlights the roles of EIF2S3Y in pluripotency maintenance and proliferation regulation of ES cells, and also could provide a valuable model to study germ cell generation and cancer development using ES cells.

## MATERIALS AND METHODS

### Cell culture and differentiation

ES cells were purchased from ATCC (SCRC-1010, Manassas, USA) and cultured in Dulbecco Modified Eagle Medium (DMEM) supplemented with 15% fetal bovine serum (Gibco, Massachusetts, USA) and 1000 U/ml leukemia inhibitory factor (Millipore, Massachusetts, USA). For ES differentiation, ES cells were cultured in the above ES medium in which leukemia inhibitory factor was replaced by 1 μM RA (Sigma-Aldrich, Missouri, USA) for 48 h.

### Generation of ES-derived cell lines

Lentivirus was generated as previously described [53]. For cell immortalization, ES cells were plated at a density of 3 × 10^5^ cells in a 35-mm dish. After 12 h, the cells were transduced with virus-containing supernatant with 10 μg/ml polybrene (Sigma-Aldrich, Missouri, USA) and incubated overnight at 37°C and 5% CO_2_. After 24 h, the medium was replaced with fresh ES medium, and the cells were cultured for more than 2 weeks with medium containing 350 ng/ml puromycin.

The screened cells were digested with TrypLE^™^ Select (Invitrogen, Massachusetts, USA) and diluted to approximately 100 cells/ml. A total of 10 μl of the cell suspension was plated onto a 96-well plate coated with 0.1% gelatin. Then, the cells were seeded at 1 cell/well using a microscope and expanded in ES medium containing 200 ng/ml puromycin.

To identify the cell lines from the monoplast above, we used a pair of primers with the sense sequence matching the lentivirus backbone and the anti-sense sequence matching the gene EIF2S3Y (forward: CAGTC AAGGCAGATTTGGGTAA, reverse: GAGCCATTTG ACTCTTTCCACA).

### Polymerase chain reaction (PCR) and quantitative real-time PCR (qRT-PCR) analysis

PCR and qRT-PCR procedures were described previously [54]. The qRT-PCR primers used in this study are listed in Supplementary Table 1.

### Immunofluorescence staining

The immunofluorescence staining was conducted as previously reported [55]. Detailed information for the antibodies are : TET1 (1:300, GeneTex, California, USA); 5hmC (1:500; Active Motif, California, USA); OCT-4 (1:300; Chemicon, Massachusetts, USA); stage-specific embryonic antigen 1 (SSEA-1; 1:200; Chemicon, Massachusetts, USA); H3K9me2 (1:500; Sino Biological Inc., Beijing, China); H3K27me3 (1:500; Sino Biological Inc., Beijing, China); Sox2 (1:200; Chemicon, Massachusetts, USA); proliferating cell nuclear antigen (PCNA; 1:200; Millipore, Massachusetts, USA); 5-bromo-2′-deoxyuridine (BrdU; 1:300; Santa Cruz, California, USA), FITC-conjugated secondary antibody (1:500; Chemicon, Massachusetts, USA), HOECHST33342 (Sigma-Aldrich, Missouri, USA). The immunofluoresence intensity was analyzed by ImageJ software (National Institutes of Health, USA).

### Cell transplantation

Male ICR mice were purchased from the animal center of the Fourth Military Medical University in Xi'an. At the age of 7w, the mice were treated with busulfan at 30 mg/kg (body weight) for 3 weeks to be rendered infertile. Approximately 3 × 10^5^ cells in PBS per testis were microinjected into the seminiferous tubules of the infertile mice through the efferent duct. For a mouse, one testis was injected with specific cells and the other one treated with PBS. After 3 weeks, all testes were harvested and fixed in 4% formaldehyde overnight for further analysis [56]. All experiments were performed following approval from the NWSUAF Animal Care and Use Committee.

### Cell proliferation assay

The cell growth curve was made according to a previous report [57].

The cell cycle assay was conducted according to the manufacturer's instructions (Cell Cycle Staining Kit, Liankebio, China), and the samples were analyzed by flow cytometry (Beckman Coulter, California, USA) [58]. The proliferation index was determined as (percentage of S phase + percentage of G2 phase)/percentage of G1 phase.

The BrdU assay procedures were described previously [53]. Information of the reagents used was listed as follows: BrdU (Sigma-Aldrich, Missouri, USA), FITC-conjugated secondary antibody (1:500; Millipore, Massachusetts, USA). To determine the level of cell proliferation, each assay was performed in triplicate, and 5 fields were randomly chosen to count the percentage of BrdU-positive cells.

### Western blot analysis

Western blot analysis procedure was described previously [55]. The primary antibodies were listed as follows: GAPDH (1:3000, Genesci, Shanghai, China); TET1 (1:500, GeneTex, California, USA); H3K9me2 (1:1000; Sino Biological Inc., Beijing, China), H3K27me3 (1:1000; Sino Biological Inc., Beijing, China), and OCT4 (1:500; Chemicon, Massachusetts, USA). Horse-radish peroxidase-conjugated anti-rabbit antibody (1:1000, Beyotime, Beijing, China) or anti-mouse antibody (1:2000, Beyotime, Beijing, China) was used as secondary antibodies.

### Statistical analyses

Two tailed Students' *t*-test was used in this study, and the data are presented as mean ± SD. Differences were considered significant when the *p* value was less than 0.05 (*P < 0.05; **P < 0.01; ***P < 0.001). All data are representative of at least 3 different experiments and were analyzed using a GraphPad Prism software (San Diego, California, USA) [53].

## SUPPLEMENTARY MATERIALS FIGURES AND TABLE


