# Supplementary material for: EIF2S3Y suppresses the pluripotency state and promotes the proliferation of mouse embryonic stem cells
Source: Oncotarget. 2016 Feb 4;7(10):11321–31. doi: 10.18632/oncotarget.7187 (PMC4905476; doi:10.18632/oncotarget.7187)
Supplement: Supplementary file 1 [file oncotarget-07-11321-s001.pdf]

EIF2S3Y suppresses the pluripotency state and promotes the proliferation of mouse embryonic stem cells

Supplementary Materials

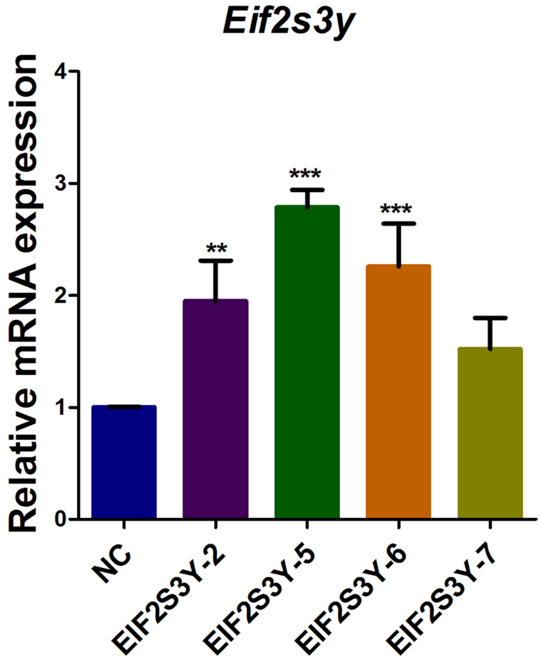

Supplementary Figure 1: *Eif2s3y* mRNA expression profile in ES-derived EIF2S3Y cell lines. Relative expression of *Eif2s3y* mRNA in NC, and EIF2S3Y-(2, 5, 6, 7) cell lines by qRT-PCR. ($n = 3$ for each group, $^{**}P < 0.01$, $^{***}P < 0.001$, vs. NC).

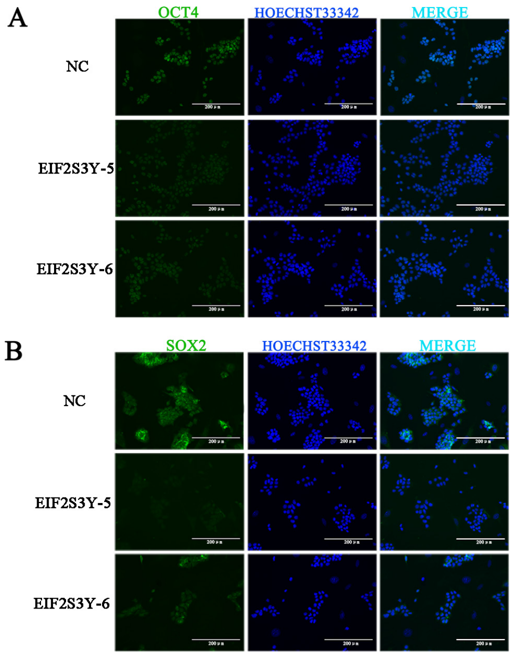

Supplementary Figure 2: Immunofluorescence staining of OCT4 and SOX2 in ES-derived EIF2S3Y cell lines. (**A, B**) Immunofluorescence staining of OCT4 (A) and SOX2 (B) (green), nucleic acids were stained with Hoechst 33342 (blue). Scale bar = 200 μm ($n = 3$ for each group).

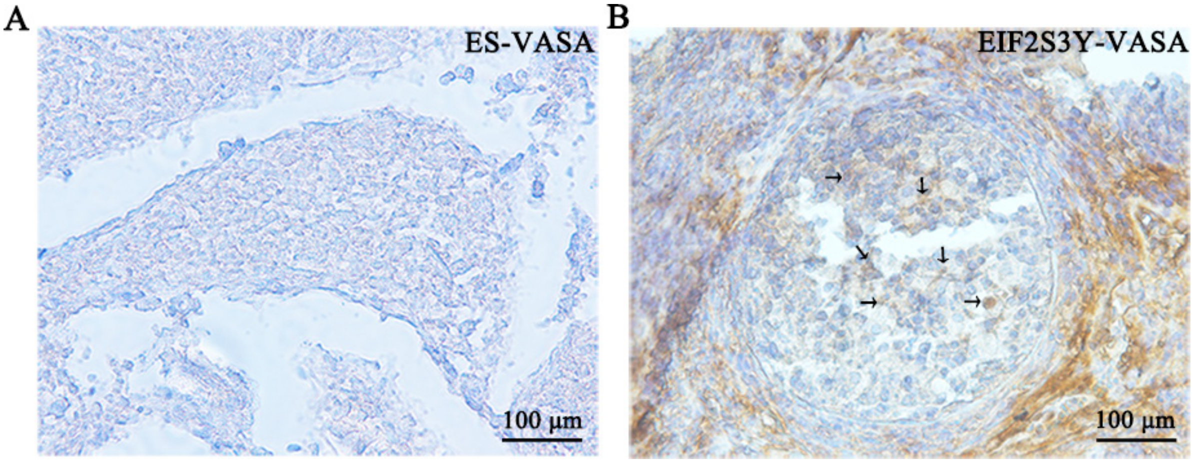

Supplementary Figure 3: Immunohistochemistry of VASA in the testes transplanted with ES-derived cell lines. (**A**) Immunohistochemistry staining of VASA in the testes that were transplanted with ES or EIF2S3Y-5 cell line. VASA positive cells were indicated with arrows. Scale bar, 100 µm (n = 2 for each group).

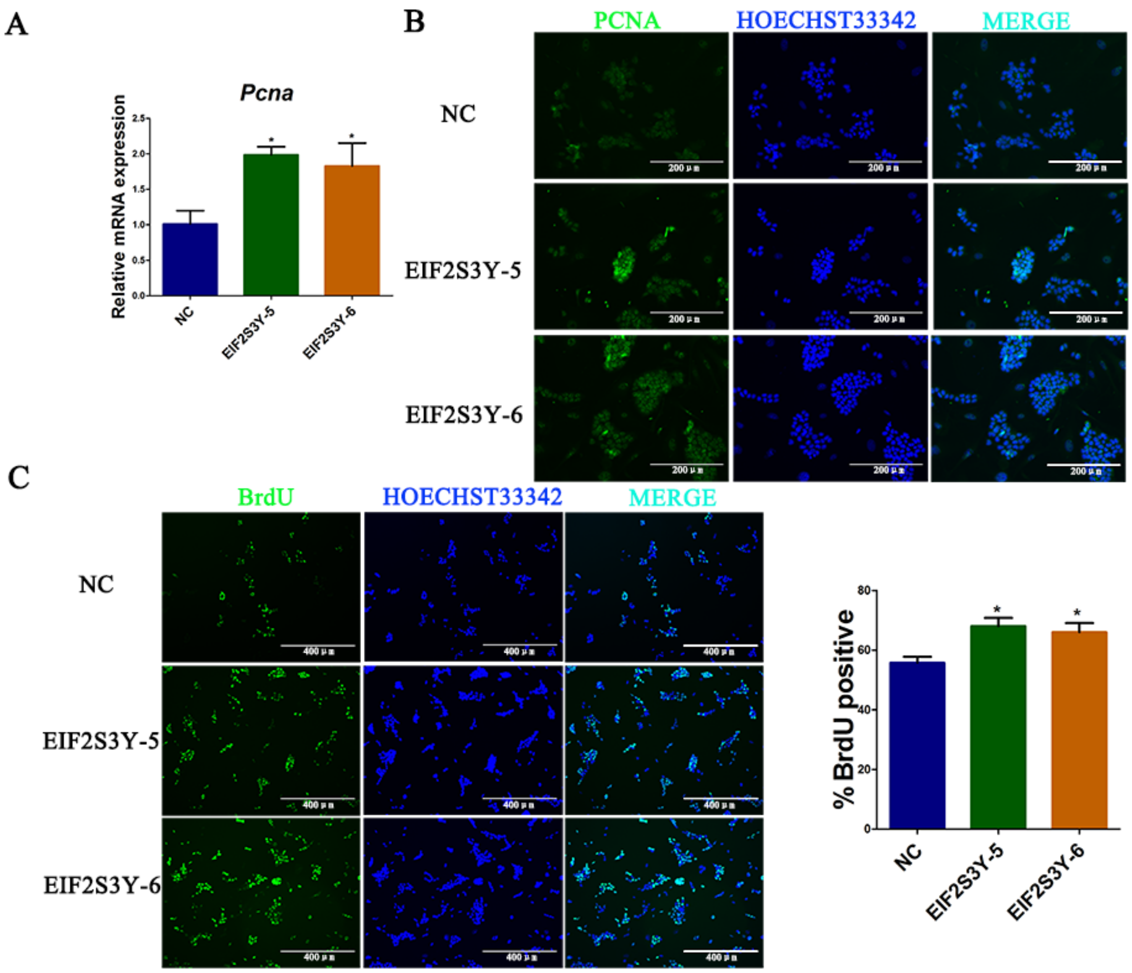

Supplementary Figure 4: Proliferation profile of ES-derived cell lines. (**A**) Relative expression of *Pcna* by qRT-PCR in NC, EIF2S3Y-5 and EIF2S3Y-6 cell lines. (**B**) PCNA immunofluorescence staining in NC, EIF2S3Y-5 and EIF2S3Y-6 cell lines. (**C**) BrdU immunofluorescence staining and percentage of BrdU-positive cells in NC, EIF2S3Y-5 and EIF2S3Y-6 cell lines. ($n \geq 3$ for each group, *$P < 0.05$, vs. NC).

Supplementary Table 1: Sequences of primers used in PCR amplification

	Primer Sequence (5′-3′)
Gapdh	Fwd: TGGCCTTCCGTGTTCCTAC
	Rev: GAGTTGCTGTTGAAGTCGCA
Eif2s3y	Fwd: ATCTTGTCCTCAACCTCAGACT
	Rev: TTCTTTAGCCTGGCTTTCTTTCA
Ssea-1	Fwd: ACGGATAAGGCGCTGGTACTA
	Rev: GGAAGCCATAGGGCACGAA
Nanog	Fwd: TTCTTGCTTACAAGGGTCTGC
	Rev: AGAGGAAGGGCGAGGAGA
Prdm1	Fwd: TGGAGGACGCTGATATGACT
	Rev: CTTACCACGCCAATAACCTC
Oct-4	Fwd: GGCGTTCTCTTTGGAAAGGTGTTC
	Rev: CTCGAACCACATCCTTCTCT
Sox2	Fwd: CATGAGAGCAAGTACTGGCAAG
	Rev: CCAACGATATCAACCTGCATGG
Klf4	Fwd: TGGTGCTTGGTGAGTTGTGG
	Rev: GCTCCCCCGTTTGGTACCTT
Pcna	Fwd: AGTGGAGAACTTGGAAATGGAA
	Rev: GAGACAGTGGAGTGGCTTTTGT
Cylin A	Fwd: TGGCTGTGAACTACATTGA
	Fwd: ACAAACTCTGCTACTTCTGG
Cylin E	Fwd: GTGGCTCCGACCTTTCAGTC
	Fwd: CACAGTCTTGTCAATCTTGGCA
